# Impact of early applied upper limb stimulation: The EXPLICIT-stroke programme design

**DOI:** 10.1186/1471-2377-8-49

**Published:** 2008-12-17

**Authors:** Gert Kwakkel, Carel GM Meskers, Erwin E van Wegen, Guus J Lankhorst, Alexander CH Geurts, Annet A van Kuijk, Eline Lindeman, Anne Visser-Meily, Erwin de Vlugt, J Hans Arendzen

**Affiliations:** 1 Dept. Rehabilitation Medicine, VU University Medical Centre, PO Box 7057, 1007 MB, Amsterdam, The Netherlands; 2 Dept. Rehabilitation Medicine, Leiden University Medical Centre, PO Box 9600, 2300 RC, Leiden, The Netherlands; 3 Dept. Rehabilitation Medicine, Radboud University Medical Centre, PO Box 9101, 6500 HB, Nijmegen, The Netherlands; 4 Dept. Rehabilitation Medicine, University Medical Centre and Rudolf Magnus Institute of Neuroscience, Heidelberglaan 100, 3584 CX, Utrecht, The Netherlands; 5 Rehabilitation Centre "De Hoogstraat", PO Box 85238, 3508 AE, Utrecht, The Netherlands; 6 Department of Biomechanical Engineering, Faculty of Mechanical Engineering, Delft University of Technology, Mekelweg 2, 2628 CD, Delft, The Netherlands

## Abstract

**Background:**

Main claims of the literature are that functional recovery of the paretic upper limb is mainly defined within the first month post stroke and that rehabilitation services should preferably be applied intensively and in a task-oriented way within this particular time window. EXplaining PLastICITy after stroke (acronym EXPLICIT-stroke) aims to explore the underlying mechanisms of post stroke upper limb recovery. Two randomized single blinded trials form the core of the programme, investigating the effects of early modified Constraint-Induced Movement Therapy (modified CIMT) and EMG-triggered Neuro-Muscular Stimulation (EMG-NMS) in patients with respectively a favourable or poor probability for recovery of dexterity.

**Methods/design:**

180 participants suffering from an acute, first-ever ischemic stroke will be recruited. Functional prognosis at the end of the first week post stroke is used to stratify patient into a poor prognosis group for upper limb recovery (N = 120, A2 project) and a group with a favourable prognosis (N = 60, A1 project). Both groups will be randomized to an experimental arm receiving respectively modified CIMT (favourable prognosis) or EMG-NMS (poor prognosis) for 3 weeks or to a control arm receiving usual care. Primary outcome variable will be the Action Research Arm Test (ARAT), assessed at 1,2,3,4,5, 8, 12 and 26 weeks post stroke. To study the impact of modified CIMT or EMG-NMS on stroke recovery mechanisms i.e. neuroplasticity, compensatory movements and upper limb neuromechanics, 60 patients randomly selected from projects A1 and A2 will undergo TMS, kinematical and haptic robotic measurements within a repeated measurement design. Additionally, 30 patients from the A1 project will undergo fMRI at baseline, 5 and 26 weeks post stroke.

**Conclusion:**

EXPLICIT stroke is a 5 year translational research programme which main aim is to investigate the effects of early applied intensive intervention for regaining dexterity and to explore the underlying mechanisms that are involved in regaining upper limb function after stroke. EXPLICIT-stroke will provide an answer to the key question whether therapy induced improvements are due to either a reduction of basic motor impairment by neural repair i.e. restitution of function and/or the use of behavioural compensation strategies i.e. substitution of function.

EXPLICIT is registered at the Netherlands Trial Register (NTR, ., TC 1424)

## Background

**Figure 1:**
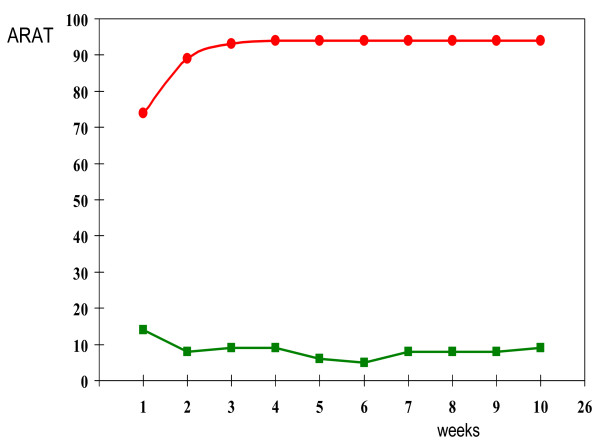
**Probability (%) of achieving dexterity (ARAT ≥10 points) at 6 months post stroke (N = 102) starting in the first week post stroke**. Based on the Fugl Meyer scores of the flaccid arm, optimal prediction of arm function outcome at 6 months could be made within 4 weeks after onset. Patients with a favorable prognosis are presented with a red line, whereas lack of voluntary motor control of the leg in the first week with no emergence of arm synergies within 4 weeks post stroke was associated with poor outcome at 6 months (green line, [5,10]).

Each year, more than 32,000 patients sustain a stroke [1]. The incidence is expected to have increased by 30–45% in 2015 [2]. About 80% of the survivors have an upper limb paresis immediately after stroke onset [3], whereas only one third of all stroke patients have regained some dexterity at 6 months [4]. Recent prospective cohort studies showed that the functional outcome at 6 months is highly predictable within a critical time window of 4 weeks post stroke [4,5]. (Figure 1.)

There is no evidence that these recovery patterns can be influenced by any specific treatment approach [6,7], though a number of studies suggest that rehabilitation should be initiated as soon as possible, preferably within the critical time window of 4 weeks post stroke [5]. In addition, treatment should be applied intensively and in a task-oriented way [4,8-10]. In particular a number of recent longitudinally conducted studies suggest that an early return of wrist and finger extension is a key factor for regaining dexterity [11-13].

Unfortunately, the underlying mechanisms responsible for recovery are not well understood [8-10,14]. A better understanding of the factors that facilitate upper limb recovery, as well as the time windows in which these recovery mechanisms work best are a prerequisite for improving our rehabilitation services in the future. Recently, several controlled trials in the chronic phase after stroke have shown that innovative therapies such as constraint-induced movement therapy (modified CIMT, [15-17]) and EMG-triggered neuromuscular electrical stimulation (EMG-NMS) [18], may improve upper limb function. So far, well designed and conducted randomized clinical trials investigating the promising effects of modified CIMT [19,20] and EMG-NMS in the acute or subacute phase after stroke are lacking. A few studies have found that improvements induced by modified CIMT [21,22] or EMG-NMS of the affected upper limb [23,24] coincide with cortical reorganizations of motor maps in patients suffering from chronic stroke. This suggests that neuroplasticity is an important mechanism for functional recovery of the upper paretic limb. However, the impact of the above-mentioned therapies on dynamics of cortical reorganization has never been investigated in stroke patients. Recent longitudinal studies also suggest that functional recovery is more than neural repair alone. For example, kinematically controlled studies showed that recovery of the paretic arm and hand strongly depends on adaptive trunk movements when distal motor deficits are present [10,25]. Therefore, understanding functional recovery of the upper limb requires not only knowledge about longitudinal changes in neuroplasticity but also about the compensation strategies patients use [10] together with changing limb neuromechanics, i.e. joint stiffness, muscle/motor function and control [26-32]. In other words, the functional impact of the time-dependent changes in cortical neuroplasticity, as well as the adaptive compensation strategies used to deal with existing motor deficits and possible neuromechanical constraints need to be explored in order to improve our knowledge about what exactly patients learn when functional improvement is observed. Therefore, the objective of the present EXPLICIT-stroke programme is to address the following research questions in the next 5 years:

(1) Is an early modified CIMT programme more effective in terms of recovery of the paretic upper limb than conventional care in stroke patients with a favourable prognosis for recovery of the upper limb? (Project A1)

(2) Is an early EMG-NMS programme for wrist and finger extensors more effective in terms of return of dexterity than conventional care in patients with initially poor prognosis for upper limb recovery? (Project A2)

(3) Are modified CIMT-induced gains reinforced by recruitment of cortical activation in the ipsilesional hemisphere when compared to usual care? (Project B1)

(4) Can improved performance of a functional reaching task be explained on the basis of behavioural compensation strategies? (Project B2)

(5) How and to what extent do wrist neuromechanical properties, i.e. stiffness, motor function and control change in the first 6 months post stroke? (Project B3)

(6) Does early intervention affect endpoint neuromechanics (Project B3)

(7) How are improvements in dexterity longitudinally related to changes observed in kinematics, neuromechanics and cortical activation (Project C).

## Methods

EXPLICIT-stroke will include 180 first ever stroke patients within five years. The study has been approved of by the Medical Ethical Reviewing Committees of the Leiden University Medical Centre (main reviewing committee: protocol number P08.035, Dutch Central Committee on Research Involving Human Subjects, CCMO, protocol number NL21396.058.08), the VU Medical Center, Amsterdam, the University Medical Centre St. Radboud, Nijmegen and the University Medical Centre Utrecht, The Netherlands.

**Figure 2:**
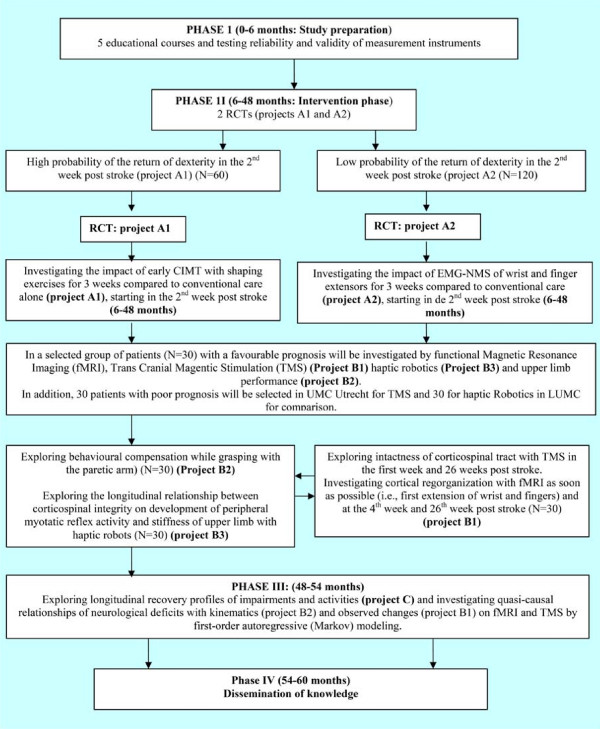
**Illustration of coherence between projects of EXPLICIT and subsequent phases**.

EXPLICIT includes 3 main projects: A, B and C. The two A projects (A1 and A2) aim to investigate the effects of modified CIMT and EMG-NMS respectively, whereas the B projects aim to examine (1) the learning- and time-dependent changes in cortical neuroplasticity (project B1); 2) the kinematic changes (project B2) and 3) the neuromechanical changes (project B3) related to upper limb recovery in the first 6 months post stroke. Within project C, longitudinal, quasi-causal relationships are being sought between upper limb function improvements (A projects) and mechanisms of post stroke recovery (B projects). A schematic representation of the EXPLICIT programme is presented in Figure 2.

### The A projects of Explicit

The 'A' projects comprise two RCTs investigating the effectiveness of early interventions on paretic upper limb recovery in a 3-week programme starting in the second week post stroke and ending in the fourth week. In the first RCT (project A1), 60 first-ever MCA stroke patients with a National Institute of Health Stroke Score, NIHSS 1 or 2 and a voluntary extension of wrist and fingers of at least 10 degrees assessed at the end of the first week post stroke will be randomized to either an experimental group undergoing task-oriented exercises i.e. shaping procedures of the upper limb, supplemented by modified constraint-induced movement therapy (modified CIMT), or a control group. All patients will be recruited in four medical centres participating in EXPLICIT, i.e. Leiden University Medical Centre, LUMC; VU University Medical Centre, VUmc; Radboud University Medical Centre, RUMC and University Medical Centre Utrecht, UMCU.

In the second RCT (project A2), 120 patients with an initially poor prognosis for recovery of dexterity at the end of the first week, i.e. NIHSS score 3 or 4 and no voluntary control of the paretic upper limb, will be recruited and randomized either to an intervention group receiving conventional care supplemented by EMG-triggered electrotherapy (EMG-NMS) to stimulate wrist and finger extensors or to a control group.

After the intake procedure, which will include the first assessment of outcome variables, we will apply separate restricted randomization procedures (in permuted blocks) with random number tables for each participating university medical centre and for each of the projects A1 and A2. Concealed allocation will be effectuated with sealed opaque envelopes. Before randomization, patients and their families will be informed that all interventions may improve outcome, but that they will remain naive with respect to the supposed efficacy of the experimental condition to which they have been allocated. Nurses, speech therapists and social workers will continue to provide regular care depending on the patients' needs.

### Project A1

#### Background and hypotheses

Claims made in the literature are that therapy should be intensive and task-oriented and that therapy is most effective in the early stages after stroke. Two research hypotheses will be addressed in project A1: 1) Acute or subacute hemiplegic stroke patients treated for 3 weeks with an intensive daily shaping programme for the paretic upper limb will show significantly less upper extremity impairment and disability after 3 weeks compared to those patients receiving conventional training; 2) The gains achieved by modified CIMT will sustain over time, in such way that these patients will show less upper extremity deficits, and hence better outcomes in terms of dexterity at 6 months post stroke when compared to those patients who received conventional care.

#### Recruitment

Sixty subjects with a favourable prognosis will be recruited. Inclusion criteria are: 1) an upper limb deficit, i.e. a NIHSS score of 1 or 2 in the second week post stroke; 2) a first-ever ischemic lesion in the territory of the MCA, verified by CT and/or MRI scan; 3) age between 18 and 80 years; 3) written or oral informed consent; 4) be able to sit for 30 seconds without support; and 4) sufficient motivation to participate.

The exclusion criteria are 1) a pacemaker or other metallic implants; 2) upper extremity orthopaedic limitations; 3) not being able to communicate i.e. < 4 points on the Utrecht Communication Observation, UCO [33]; 4) a Mini Mental State Examination, MMSE score of 22 points or less, [8] and 5) recombinant tissue plasminogen activator (rTPA, or alteplase) treatment. There will be no restrictions with respect to age, ethnicity or socio-economic status.

#### Design and intervention

In the modified CIMT trial, patients in the experimental group will receive 30 minutes of task-oriented training of the upper extremity, aimed at improving dexterity of the paretic arm (i.e. shaping) for 5 days a week over 3 consecutive weeks. Task difficulty will be progressively increased using behavioural techniques of shaping and successive approximation [34]. In addition, the Padded Safety Mitt (Sammons Preston # 6727; Sammons Preston, Inc, Bolingbrook, IL, USA) will be applied for 2 hours to immobilize the non-paretic arm following each treatment session (excluding weekends). During toileting and under other circumstances that require safe balance, patients will be allowed to remove the padded safety mitt. The content and duration of the modified CIMT therapy as well as the shaping exercises will be recorded in a patients' log reflecting different treatment goals. Conventional care will consist of exercise therapy based on the principles of the NDT concept for a restricted one-by-one intervention time of 30 minutes per working day, 5 days a week for 3 consecutive weeks excluding weekends. Content and daily duration of conventional therapy will be recorded in the patient logs using different treatment codes for exercise intervention types and intervention goals (See for example Kwakkel et al [8]). The modified CIMT programme is focused on regaining voluntary wrist and finger extension of the paretic hand by intensive exercise therapy 2 times a day 30 minutes for 3 weeks. The protocol for modified CIMT is available at .

All therapy sessions will be individually applied by a local trained physical therapist (PT) and/or occupational therapist (OT) working in one of the participating university medical centres, or, if a patient is discharged earlier, in a regional rehabilitation centre.

After 3 weeks, the intensity of the interventions implemented by the OTs and PTs will be standardized in both arms of project A1 and restricted to exercise therapy alone, given for a maximum of 30 minutes per working day. Duration and aims of interventions implemented by PTs or OTs will be monitored by means of monthly phone calls by the local member of the project staff at the university medical centre.

#### Power analysis of project A1

Analysis is based on a statistical power of 80% with an alpha of 5% for detecting a meaningful difference of 6 points i.e. 10% on the Action Research Arm Test, ARAT sum score as the primary outcome measure [35]. The estimated population variance is based on the outcome of a randomized clinical trial in 102 stroke patients with a first-ever MCA stroke i.e. a standard deviation of 8 points measured with ARAT at 2 weeks post stroke [5,8]. Controlling for dependency (test-retest reliability of 0.9 for ARAT based on 5 repeated measurements in the first 5 weeks), a sample-size of 60 patients including 10% drop outs should be sufficient.

### Project A2

#### Background and hypotheses

Patients with a poor prognosis for functional recovery (estimated to include about 2/3 of all stroke patients with a MCA stroke) will undergo an intensive stimulation programme of wrist and finger extensors using EMG-triggered neuromuscular stimulation (EMG-NMS). These patients are unable to move their paretic upper limb voluntarily. Return of voluntary motor control can so far only be facilitated by somatosensory input and imaginary movement. The assumed positive impact of EMG-NMS is based on the finding that 2 hours of sensory stimulation of the median nerve of a paretic limb may result in improved pinch strength [23]. This improvement in muscle strength correlates with the stimulus intensity and has been identified in the absence of motor training. Virtual imaging has also been found to improve cortical plasticity and motor performance of the upper limb in chronic stroke [24]. The above results suggest that somatosensory stimulation, combined with virtual imaging by applying EMG-NMS, may be a promising adjuvant therapy to initiate active motor control in stroke patients with an upper limb deficit. It is hypothesized that cortical ipsi-lesional and contra-lesional plasticity is facilitated by EMG-NMS of wrist and finger extensors especially early after stroke, when the outcome in terms of dexterity has not yet been defined, hence increasing the probability of return of some functional dexterity [24]. In addition, it is believed that the movement imagery component causes neural networks in the motor cortex, associated with the specific imagined movement, to be stimulated and reinforced. Evidence supporting this theory has been found using fMRI [24]. Based on this 'use it or lose it' principle [36-38], one may assume that a higher percentage of patients in the experimental group will regain some dexterity in terms of ARAT scores, i.e. > 9 points [5], when compared to the control group. If triggering with the paretic limb proves to be not possible, EMG activity of the non-paretic wrist and fingers extensors will be used to trigger activation of the paretic limb, since ipsilateral activation is also known to reinforce cortical activity in the affected hemisphere after stroke [39].

#### Recruitment for project A2

A total of 120 subjects with a first-ever MCA stroke and an initially poor prognosis for recovery of dexterity (NIHSS score 3 and 4) in the second week post stroke will be recruited. Inclusion and exclusion criteria will be identical as for project A1.

#### Design and intervention of project A2

In the second week post stroke, patients with an initially poor functional prognosis who have been allocated to the control group will engage in passive range-of-motion exercise and facilitation of voluntary movements for 30 minutes per week day, whereas the experimental group will receive EMG-triggered NMS (Stiwell Med 4, Otto Bock Healthcare Products GmbH, Vienna, Austria) of wrist and finger extensors each day for 3 periods of 30 minutes per working day during 3 consecutive weeks. The cyclic stimulation will be biphasic, with a pulse width of 200 μsec and intensity up to 29 mA with a frequency of 50 Hz. Patients are asked to imagine the dorsiflexing movement of fingers and wrist on the paretic side. This causes some initial muscle activity which, when detected by the EMG electrodes, amplified and presented once again to the muscle, may elicit a genuine contraction of the wrist extensors [40]. For those patients showing no EMG activity, the extensors of the non-paretic hand will be used as a trigger to activate the paretic hand. This new mode of electrical stimulation has been claimed to be superior to other types of electrical stimulation programme [18,40]. Finally, patients may learn to facilitate extensor activitity of wrist and fingers by simultaneously abducting their paretic arm (often described as Soques phenomenon). Details of the EMG-NMS treatment protocol is available at .

The intensity and content of the EMG-NMS as well as other therapies applied will be recorded in a patient log-book. Both groups will receive usual care in terms of lower limb training. After 3 weeks, usual care will be restricted to a maximum of 30 minutes of upper limb practice, monitored by monthly phone calls by the project staff. All therapy sessions will be individually applied by local physical (PT) and/or occupational (OT) therapists working at the local setting (UMC and local rehabilitation centre) where patients have been admitted. For this latter purpose, PTs and OTs who participate in EXPLICIT will have a training course in the preparation phase.

#### Power analysis for project A2

Based on a previous cohort study with 101 first-ever MCA strokes [8], patients with a poor prognosis have a probability of about 6% to regain some dexterity (ARAT > 9 points) at 6 months post stroke [8]. In project A2 we assume a proportional difference of 12% in favour of the experimental group. Based on a statistical power of 80% with an alpha of 5% and a test-retest reliability of 0.9 for ARAT with 5 repeated measurements, 120 patients should be enough to show statistical significance in favour of EMG-NMS in the poor prognosis group.

#### Outcome variables of projects A1 and A2

Primary outcome variable for both A1 and A2 projects will be the Action Research Arm test score (ARAT). Secondary outcome variables are the scores of: Motricity Index (MI) of arm and leg, Fugl-Meyer for the arm (FM-arm), Erasmus modification of the Nottingham Sensory Assessment (EmNSA), Letter Cancellation Task (LCT), Nine Hole Peg test (NHPT), a structured participant interview of real arm use using the Motor Activity Log (MAL), Stroke Impact Scale (SIS version 3.0) and Nottingham Extended ADL (NEADL). With the exception of the NEADL and SIS, all assessments will be applied to all 180 patients weekly in the first 5 weeks post stroke. Subsequently, follow-up assessments will be made at 8, 12 and 26 weeks after stroke.

The ARAT test is a performance test which assesses the ability to perform gross movements and the ability to grasp, move and release objects differing in size, weight and shape [41]. The original test consists of 19 items, rated on 4-point ordinal scales (0 to 3). By removing 4 items, a hierarchical 1-dimensional scale has been constructed [42]. The ARA test has been shown to be valid, reliable and responsive [35]. The minimal clinically important difference (MCID) will be set at about 10% of the range of the scale, i.e. 6 points [15]. For project A2, return of dexterity will be defined as 10 points or more on the ARAT [5].

The Motricity index (MI) reliably and validly assesses the presence of paresis in stroke patients by testing 6 functions rated from 0 to 100 points for each limb [8,43].

The FM-arm score is a reliable and valid motor performance test [44,45] and evaluates the ability to make movements outside the synergistic pattern.

A translated and adapted version of the MAL will be used [46] which contains the 14 original activities, 11 additional activities, and 1 optional activity chosen by the patient. Reliability and validity of the MAL has been proved in a number of studies [46]. The MAL will be administered to each applicant and, if available, their caregivers. It will be used to independently rate how well (11-point Quality of Movement [QOM] scale) and how much (11-point amount-of-use [AOU] scale) the paretic arm was used spontaneously to accomplish 30 activities of daily living outside the laboratory [47].

The EmNSA is a 3 point ordinal scale measuring sharp-blunt discrimination, two-point discrimination and proprioception. With exception of the two-point discrimination, intra- and inter rater reliability are good to excellent (Kappa: 0.58 to 1.00, [48]).

The Nine Hole Peg Test (NHPT) is a reliable and valid test that measures manual dexterity [49,50] by measuring the speed with which a patient grasps and inserts (and removes) 9 pegs into a grid of vertical holes. The test will be discontinued after 150 seconds if the patient is still unable to insert any pegs. Reliability and validity have been assessed and norms are available [49,50].

Visual inattention will be evaluated by the letter cancellation test which has shown high test-retest reliability, ranging from 0.78 to 0.90 (p < 0.001) for the number of omissions on the sound and neglected field sides respectively [8].

The arm-hand domain of the Stroke Impact Scale (SIS, version 3.0) will be used to evaluate patients' perceived outcome for the paretic upper limb. Version 3.0 of the SIS is a full-spectrum health status interview that measures changes in 8 impairments, function and quality of life subdomains following stroke [51]. Each domain will be analysed separately. The upper limb part of the SIS includes 5 questions about patients' perceived competency to keep their balance, to transfer, to walk in the house and negotiate stairs, to get in and out of a car and to move about in their own community. Each item is scored from 'not difficult at all' to 'cannot do at all' on a 5-point rating scale. A difference of 5 points (10%) on the 'hand function' domain of the SIS is perceived as clinically relevant [17]. The SIS has shown excellent clinimetric properties in terms of concurrent and construct validity, test-retest reliability and responsiveness [52,53].

The Nottingham Extended ADL scale is based on a self-report questionnaire about levels of activity actually performed [54]. The scale has proved to have reasonable hierarchical (ordinal) properties in stroke patients [55].

#### The B projects of Explicit

The effect of modified CIMT on the process of neurological recovery is not fully understood. So far, it is assumed that the impairment of hand function is worsened by learned non-use and that this leads to a loss of cortical representation of the upper limb [37]. It is claimed that these processes can be reversed by constraining the unaffected limb, combined with intensive practice i.e. shaping of the paretic hand. However, the theory of learned non-use has been called into question and there is uncertainty about the nature of the improvements induced by modified CIMT, although some evidence exists that modified CIMT increases spontaneous use of the hand by recovery of existing deficits, either through reduction of learned non-use or by overcoming the sense of effort during movement [37]. In order to improve our understanding of mechanisms that may underlie functional recovery and therapy-induced effects our second objective will be to investigate the CIMT-induced plastic changes in the affected and non-affected hemispheres by fMRI and TMS and compare these with a control group (Project B1). To this end, 30 patients will be selected from project A1 viz. 15 from the experimental and 15 from the control group.

Although it is assumed that modified CIMT can improve dexterity in the acute or subacute phase, no studies have so far analysed in detail whether this improvement reflects reduction of basic motor impairments or works by learning compensatory movement strategies (project B2). Finally, understanding the observed adaptive behavioural movement strategies requires that the neuromechanical changes of the musculoskeletal system are assessed. For this purpose, it is of vital importance to understand the relationship between parameters characterizing CNS function (such as TMS and fMRI) and parameters describing end-point neuromechanical behaviour (project B3).

#### Recruitment for the B projects

For the B projects 30 subjects from project A1 with an initially favourable prognosis within the first week post stroke will be recruited to undergo fMRI, TMS, kinematical measurements and haptic robotics. As a control group, 30 patients with an initially poor prognosis (i.e. from project A2) will be recruited for TMS (project B1) and haptic robotics.

#### Power analysis

Concerning fMRI, calculation is based on measures of Brodmann areas 9 and 46 in which the fMRI activation is assumed to be more difficult than in primary motor and sensory cortices, thus, a conservative estimate is obtained. The mean increment of the intensity of the signal in the volumes of interest (VOI 's, %) and the standard deviation of the normal signal intensity have both been found to be 1.5, whereas the normalized minimal difference between groups that is detectable with fMRI is estimated to be 1.15. Calculation of the required sample size is based on the formula {N = [2*(z(1-α/2)-z(1-β)) raise to square*(var(n)/Δ)] in which α and β are set at 0.01 and 0.10, respectively. This leads to a sample size of 13 patients in both the control and experimental groups. Based on an expected drop-out rate of 10%, we would need at least 30 stroke victims. It has been calculated that this number will also guarantee sufficient power for projects B2 and B3

### Project B1

#### Background and hypotheses

In study B1, it is hypothesized that the strategy of motor re-mapping after injury of the primary motor cortex M1 of the hand will mainly depend on the extent and severity of Wallerian degeneration of the pyramidal tract. Only in subjects who show some preservation of M1 will TMS be associated with a return of dexterity at 6 months [10,55,56]. Contra-lesional recruitment of secondary motor areas in the non-involved hemisphere after stroke is considered to reflect compensation strategies that are enhanced by application of modified CIMT, compared to those victims who receive conventional care. In line with this hypothesis, it is further expected that (persistent) recruitment of corresponding contra-lesional secondary motor areas, including primary (M1) cortex, supplementary motor cortex (SMA), cingulate cortex (CC) and dorsolateral (dPMA) and ventral pre-motor areas (vPMA), beyond 4 weeks will be associated with poor quality of upper limb recovery and high use of compensation strategies. Accordingly, it is expected that patients demonstrating normalization of upper limb function will show a concomitant reduction of contra-lesional cortical activation on fMRI and a shift of activation back to the original M1, SMA and PMA areas of the affected hemisphere. In those patients who fail to show substantial recovery to normal, compensatory recruitment of the non-affected hemisphere will persist. In line with this hypothesis, we assume that early applied modified CIMT will be associated with a predominantly increased ipsi-lesional recruitment of activity of M1, SMA, dorsal and ventral PMA when compared to those patients who received the control treatment. Higher lateralization indexes will then be found in volumes of interest (VOI's). Therefore, low impairment recovery profiles and high compensatory movement strategies i.e., bending and rotating the trunk in a standardized reaching task, are expected to be positively associated with contra-lesional activity patterns and low lateralization indexes. Contrastingly, patients exhibiting behavioural normalization of upper limb performance in the standardized reaching task will show focusing of activity towards the physiologically recruited areas i.e., M1, SMA and PMA as VOI' s in the ipsi-lesional hemisphere, after a possible initial recruitment of contra-lesional areas.

#### Design

**Figure 3:**
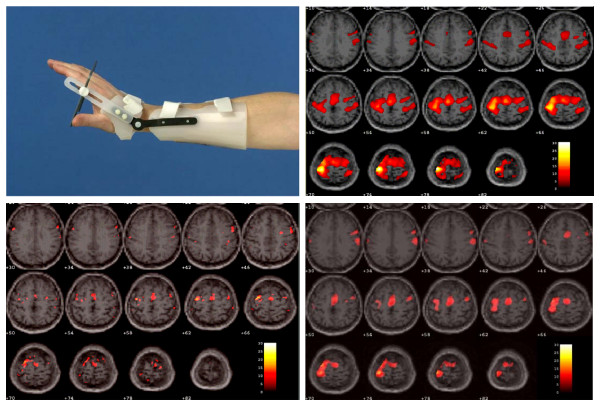
**a-d Illustration of the orthotic hand-device for B1, fMRI project (Figure 3a)**. Preliminary results from a 42 years healthy subject are shown in figures 3b-d. First, the subject was asked to perform the motor extension paradigm actively (Figure 3b). Similarly, 10 runs of 10 passive movements were executed by pushing the orthotic device manually (Figure 3c). Finally, the subject was asked to perform the task imaginary with the same block design, while keeping both arms relaxed (Figure 3d).

fMRI data will be acquired with a Philips ACS-NT 1.5 tesla clinical scanner, using the blood oxygen level-dependent (BOLD) sensitive, navigated 3D PRESTO pulse sequence [57]. Parameter settings will be: TE (echo time) 35 ms, TR (repetition time) 24 ms, flip angle 9 degrees, FOV (Field of View) 180 × 225 × 91 mm, matrix 52 × 64 × 26, voxel size 3.51 mm isotropic, scan time per fMRI volume 2.4 seconds. Following the fMRI procedure, an anatomical scan will be acquired with a slice thickness of 1.2 mm. Although fMRI does not allow direct measurement of neural activity, activation-induced BOLD signal changes occur in agreement with known functional topography. fMRI measurements will be obtained as soon as patients are able to extent their wrist beyond 10 and their fingers beyond 20 degrees. The second and third measurements will be made at the fifth week and at 6 months post stroke. In the fMRI experiments, 3 different motor tasks, in blocked design (active extension of wrist and fingers, passive extension and imaginary extension) will be executed in random order. First, the fMRI protocol will be applied in 5 healthy, age-matched subjects. Before fMRI scanning, patients will be trained to perform active extension movements with the wrist and fingers, using a wrist-hand orthosis which will guarantee a correct movement in the flexion-extension direction. In addition, the same movement will be executed passively and as an imaginary movement during the fMRI scanning. Before each fMRI study, EMG silence will be controlled during a sham fMRI training session using biofeedback electrodes attached to the extensors and flexors of the paretic and non-paretic hands. A metronome will be used to maintain the 0.3 Hz rhythm. Activation patterns will be analysed with statistical Parametric Mapping (SPM2) software. Voxels will be considered active if the task-related t-value exceeds 4.0, corresponding to p < 0.05, Bonferroni-corrected for the total number of voxels in all VOI's. Preliminary results from a 42-year-old healthy subject are shown in Figure 3. The fMRI scanning time is estimated to take about 50 minutes.

To control for the severity of motor deficit of the corticospinal tract (M1) in the involved and non-involved hemispheres, Motor Evoked Potentials (MEP) will be recorded within the first days post stroke and will be repeated 6 months after. The mechanisms of Transcranial Magnetic Stimulation (TMS) – induced changes in muscle activity (using a Magstim 200 stimulator) have been shown to involve both direct corticospinal and transsynaptic projections. The intensity of stimulation will be slowly increased until the stimulation induces visible muscle twitches in the bellies of the abductor digiti minimi muscle and biceps brachii muscle [58]. These muscles will be regarded as representative of proximal and distal motor functions in the upper extremity. Additionally, extensors of wrist and fingers (i.e., mm. extensor carpi radialis longus and mm. extensor digitorum) will be tested. Bipolar electromyographic (EMG) recordings will be obtained from pairs of surface electrodes using a Nicolet Viking or Oxford Synergy electromyograph [59-61]. The amplitude, latency and area under the curve will be used to quantify the MEP. In addition, MEP parameters i.e. motor thresholds and silent periods as a reflection of intracortical inhibition found on the paretic side will be compared with those on the non-paretic side as well as with 10 healthy age- and gender-matched controls [62].

### Project B2

#### Background and hypothesis

There are strong indications that motor recovery after cortical injury occurs to a large extent through behavioural compensation strategies [10,36,63]. These can be considered to be potential confounders in our understanding of 'neural repair' and related cerebral recovery after stroke [10,36]. For example, in one recent study of stroke patients, moderately to severely impaired subjects used compensatory strategies of the trunk to accomplish a pointing task, rather than trying to achieve restitution of the original arm function [25,64]. Cortical changes detected by fMRI may therefore reflect alternative (compensatory) motor skill learning rather than restoration of representations lost due to the infarct or the non-use of the limb. As a result, these studies failed to differentiate between 'true motor recovery' and 'compensatory motor recovery'. This has caused considerable difficulty in the interpretation of such changes in fMRI studies, apart from other potential confounding factors such as a lack of control over mirror movements, strength, precision and the timing of imaginary movements (motor paradigm) during fMRI scanning.

#### Design

The performance of the upper limb will be assessed using a standardized reaching task. Measurements will start as soon as the patient is able to execute the reaching task on the ARAT. Measurements will be repeated weekly until the fourth week post stroke, and at 8 and 26 weeks post stroke. One 10 cubic cm block weighing 200 g will be positioned on the top of an ARAT box in front of the subjects, at eye level. Patients will be asked to pick up the block with the paretic arm while sitting in front of the ARAT box. The distance between block and body will be standardized by taking subject's arm's length as the maximum reaching distance. Subjects will be asked to keep their trunk positioned against the back of their chair during the reaching task. However, if the reaching task cannot be performed, compensatory bending of the trunk will be allowed. At the start of each attempt, the patient's hand rests on a pressure-sensitive switch which is positioned on the floor of the ARAT box. The event of picking up the block will be registered by another pressure-sensitive switch, which is positioned under the block on the ARAT box. Each reaching task will be executed in a preferred and comfortable manner by the patient and will be repeated at least 10 times. Between attempts, one minute of rest will be inserted in order to prevent fatigue. Kinematics will be measured using a 6 Degrees Of Freedom electromagnetic tracking device (Polhemus Liberty, Polhemus, Vermont, USA), capable of separately assessing trunk, shoulder, elbow and finger positions and rotations. Bending of the trunk and rotation of the shoulder towards the object will be regarded a indicator of compensation. Interjoint cross-correlation coefficients will be used as a reflection of synergistic dependent and independent movement control of the upper limb [64,65].

### Project B3

#### Background and hypotheses

Project B3 will investigate the relationship between CNS function and parameters describing end-point neuromechanics. It is likely that the absence or presence of corticospinal tract as established by TMS, as well as cortical reorganization as established by fMRI will go with higher joint stiffness, impaired motor function i.e. paresis and control i.e. inappropriate reflex settings or modulation. In parallel with the expected negative correlation between regaining dexterity on the one hand and persistent cortical reorganization and the absence of corticospinal tract (as established by TMS) on the other, it is hypothesized that a high degree of stiffness, paresis and absence of reflex modulation will be related to poor outcome. The longitudinal relationship between cortical and neuromechanical parameters is expected to elucidate whether peripheral neuromechanics are an independent variable in stroke recovery.

#### Design

Neuromechanics will be assessed under both passive and active conditions, using haptic robots ("Wristalyzer", Moog FCS Inc., Nieuw Vennep, The Netherlands). These are powerful, force controlled manipulators by which force- and position perturbations can be applied in a precise way to human joints while reactions of the subject in terms of forces and joint angles can be measured [30]. To apply torque perturbations, a haptic controller is used which replaces the dynamics of the real manipulator with virtual dynamics, in this case an (rotational) inertia-spring-damper system. This means that to the subject the manipulator i.e. the external environment 'feels' like a mass-spring-damper system (haptic is Greek for 'touch sense'). Previous research showed that under an active posture task primarily the negative signs of post stroke limb dysfunction dominate, i.e. the paresis component and the absence of optimal adaptation of reflexes to the environmental conditions, which is normally found [26,29,31]. As the spinal reflex loop is under control of the higher brain areas, it is likely that the peripheral spinal reflex loop properties and therefore endpoint mechanical joint behaviour is related to integrity of the corticospinal tract and the amount of reorganization of cortical brain areas. Loss of corticospinal tract integrity and persistent CNS reorganization is anticipated to be related to high joint stiffness, loss of motor function i.e. paresis and absence of reflex modulation. This combination is labelled an unfavourable cluster, while in the favourable cluster, opposite findings are linked to corticospinal tract integrity and absent or disappearing cortical reorganization. Distinguishing these clusters and their dynamics as a function of elapsing time after stroke and their relationship to upper limb function will be a significant step forward in understanding the effect of primary cortical damage on peripheral motor function. By studying the effect of CMIT and EMG-NMS, it can be estimated whether these clusters can be modified by interventions. For project B3, parameters will be measured repetitively in a cohort of N = 30 patients with an unfavourable prognosis, of which N = 15 patients will receive additional EMG-NMS therapy and N = 30 with a favourable prognosis, of which N = 15 patients will receive additional modified CIMT therapy.

### The C project

#### Background and hypothesis

The aim of project C of EXPLICIT is to discover the longitudinal association between the time-dependent cortical changes revealed by fMRI and TMS (project B1), observed changes in kinematics (project B2) and observed changes in neuromechanics (project B3) on the one hand and the improvements in dexterity found during the first 6 months after stroke on the other. This final project hypothesizes that relatively poor recovery profiles in terms of neurological deficits (FM-score, MI-arm score, somatosensory deficits and neglect) and ARAT are significantly positively associated with: 1) the use of compensation strategies expressed as bending and rotating the trunk towards an object, 2) the amount of contra-lesional activation in fMRI and TMS i.e., recruitment of cerebral activity and 3) high joint stiffness, persistent paresis and impaired reflex modulation. Those patients showing a normalization of ARAT outcome i.e. > 53 points are expected to show: 1) normalization of kinematics, 2) focusing i.e. reduction of cortical activity on the original physiological areas involved and intactness of corticospinal tract on TMS and 3) normalisation of joint stiffness, motor function and control.

#### Design

The dynamics i.e. longitudinal associations will be identified by applying a first-order autoregressive random coefficient model. This statistical model of change scores enables exploration of the quasi-causal relationships between improvements in upper limb function on the one hand and neurophysiological changes (project B1), changes in body functions (including kinematics, project B2) and body structures (including neuromechanics; project B3) on the other. The random coefficient analysis will be performed with MLwin version 2.02. [66]. The iterative generalized least-squares algorithm will be used to estimate the regression coefficients. Before conducting the random coefficient analysis, the changes between successive measurements of the time-dependent covariates will be calculated and plotted to check for compliance with model assumptions. Because time constitutes an independent covariate, random coefficient analysis enables longitudinal analysis of unequally spaced time points of measurement, and is able to deal with missing values. To investigate the possible longitudinal association between upper limb function and potential covariates, bivariate longitudinal regression analysis will initially be conducted, including ARAT change scores and time-independent covariates at baseline, such as age, gender, and lateralization of stroke, as well as one time lag change scores of the time-dependent covariates such as MI-arm, Fugl-Meyer arm and LCT, as well as change scores on the laterality index of VOI in fMRI and TMS change scores. Subsequently, standardized regression coefficients will be calculated, and a multivariate regression model to predict functional recovery of dexterity based on ARAT scores will be developed. The likelihood ratio test will be used to evaluate the need to allow random regression coefficients into the model, whereas the Wald test will be used to obtain a P value for a particular regression coefficient. (See for recent examples [67,68])

## Discussion

The various projects in the EXPLICIT programme will have a major impact on treatment policies for stroke victims, acknowledging in particular that about 80% of all stroke survivors show upper limb paresis immediate after stroke [3], whereas only one third of all stroke patients have regained some dexterity at 6 months. A poor outcome of the upper limb is considered a major threat to perceived quality of life in patients suffering from stroke [4]. EXPLICIT intends to assess not only the effectiveness of early Modified CIMT and EMG-NMS for regaining dexterity, but also to examine the observed time-dependent dynamics in cortical plasticity (i.e. learning-dependent neural plasticity) and its effects on motor control. For example, EXPLICIT intends to answer the question whether therapy-induced improvements are due to a reduction of basic motor impairment by true repair (i.e. restitution of function) or to patients learning to apply compensatory movement strategies (i.e., substitution of function). This issue is perceived as a key question in the attempts to improve rehabilitation services for patients with an upper limb deficit after stroke in the near future. The 'EXPLICIT' programme is based on the hypothesis that therapy-induced cortical changes are best facilitated within the first days and weeks after stroke onset. [10,14,55,56]. EXPLICIT intends to reveal the effectiveness of early activation programmes after stroke by 1) forcing patients with a favourable prognosis to use their upper limb in a functional manner or 2) electrical stimulation of the distal paretic hand muscles in patients with an initially unfavourable prognosis for upper limb function [5]. Since 2006, meta-analyses have shown that constraining the non-paretic arm (Modified CIMT) in order to force the paretic arm into active involvement, combined with a functional exercise programme facilitating functional use, is an effective way to improve upper limb function in chronic stroke patients [17]. However, high quality RCTs started in the acute or subacute phase post stroke are lacking in the literature [6]. This makes the EXPLICIT programme innovative and unique in the field of stroke rehabilitation science. The EXPLICIT programme is based upon two hypotheses: 1) Patients with a favourable prognosis after stroke will benefit more from early Modified CIMT in terms of regaining upper limb function than from a conventional exercise programme; 2) in patients with an initial low probability of achieving some dexterity at 6 months [5], an early sensorimotor stimulation programme by means of EMG-triggered neuromuscular stimulation (EMG-NMS) of wrist and hand extensors may increase the likelihood of return of dexterity compared to an equally intensive conventional training programme. To date, the literature offers no controlled studies targeting this subgroup of patients with an unfavourable prognosis, whereas about 62% of all patients show a poor outcome of the upper limb function after 6 months [5,6] (Relevance of project A2 of EXPLICIT.)

Both projects (i.e., A1 and A2) are particularly relevant since the functional outcome of the paretic upper limb is largely defined after the first month post stroke [4,5]. In addition, the projects will investigate the predictive value of TMS [58-62] and the clinical significance of changing fMRI activity [55,56] in relation to functional recovery of the paretic upper limb [4,10] (Relevance of project B1 of EXPLICIT). In addition, it is not known how patients learn to deal with their reduced ability to modulate and coordinate the upper limb after stroke and what role compensation strategies play in this. (Relevance of project B2 of EXPLICIT). Finally, it is unknown how changes in stiffness and spinal reflex properties interfere with upper limb recovery (Relevance of project B3 of EXPLICIT). A better understanding of the neurobiological drivers of functional recovery will allow clinicians to define both the optimal treatment strategy and its time window in rehabilitation services after stroke in the near future. In fact, the knowledge produced by the projects in the EXPLICIT-stroke programme will further underpin the concepts of motor recovery and motor learning in stroke rehabilitation by addressing the key question: what exactly do patients learn when functional improvement is observed after stroke? Therefore, EXPLICIT may serve as a template for understanding functional recovery by elucidating the longitudinal relationship between the body functions, body structures, activities and participation of International Classification of Functioning (ICF, relevance project C of EXPLICIT).

Answers to different A, B and C project are expected at the end of 2012.

## Competing interests

The authors declare that they have no competing interests.

## Authors' contributions

GK: designed the EXPLICIT-programme, did execute pre-experiments and developed treatment protocols for CIMT and EMG-NMS and prepared the first draft of the paper. CM: edited the manuscript and holds primary responsibility for the B3 project. EW: revised the manuscript critically and holds primary responsibility for the B2 project. GL: revised the manuscript critically. AG: revised the manuscript critically and holds final responsibility for the B1, TMS project. AK revised the manuscript critically and holds primary responsibility for the B1, TMS Project. EL: revised the manuscript critically and holds final responsibility for the B1, fMRI project. AV: revised the manuscript critically. EV: revised the manuscript critically and holds primary responsibility for the B3 project. JA: revised the manuscript critically and holds final responsibility for the B3 project.

All authors read and approved of the manuscript

## Pre-publication history

The pre-publication history for this paper can be accessed here:


